# Knowledge and attitudes towards rotavirus diarrhea and the vaccine amongst healthcare providers in Yogyakarta Indonesia

**DOI:** 10.1186/s12913-015-1187-3

**Published:** 2015-11-30

**Authors:** Holly Seale, Mei Neni Sitaresmi, Jarir Atthobari, Anita E. Heywood, Rajneesh Kaur, Raina C. MacIntyre, Yati Soenarto, Retna Siwi Padmawati

**Affiliations:** School of Public Health & Community Medicine, Faculty of Medicine, University of New South Wales, Level 2, Samuels Building, Sydney, 2052 Australia; Faculty of Medicine, Universitas Gadjah Mada (UGM), Yogyakarta, Indonesia

**Keywords:** Rotavirus diarrhea, Vaccination, Healthcare providers, Knowledge and attitudes

## Abstract

**Background:**

Rotavirus has been identified as the most common pathogen associated with severe diarrhoea. Two effective vaccines against the pathogen have been licensed. However, many countries including Indonesia have yet to introduce the vaccine into their national immunisation programs. This study aimed to examine the attitudes of healthcare providers (HCPs) and other health stakeholders towards the pathogen and the vaccine.

**Methods:**

Semi-structured in-depth interviews were undertaken in two districts of Yogyakarta Province, Indonesia with nurses, midwives, primary care providers, pediatricians and other health stakeholders. Thematic analysis was undertaken.

**Results:**

Fourteen interviews were conducted between August and October 2013. We identified that while participants do not consider diarrhea to be an important problem in Indonesia, they do acknowledge that it can be serious if not properly treated. While the majority had some level of knowledge about rotavirus, not all participants knew that a vaccine was available. There were mixed feelings towards the need for the vaccine. Some felt that the vaccine is not ranked as a priority as it is not listed on the national program. However, others agreed there is a rationale for its use in Indonesia. The cost of the vaccine (when sold in the private sector) was perceived to be the primary barrier impacting on its use.

**Conclusions:**

The high cost and the low priority given to this vaccine by the public health authorities are the biggest obstacles impacting on the acceptance of this vaccine in Indonesia. HCPs need to be reminded of the burden of disease associated with rotavirus. In addition, reminding providers about the costs associated with treating severe cases versus the costs associated with prevention may assist with improving the acceptance of HCPs towards the vaccine. Promotion campaigns need to target the range of HCPs involved in the provision of care to infants and pregnant women.

## Background

Since the availability of the rotavirus vaccines, data on the burden of rotavirus diarrhea and the efficacy of the vaccines in low and middle-income settings has increased, highlighting the positive impact of vaccination on severe rotavirus disease. Following the introduction of the vaccine, substantial declines in rotavirus and all-cause diarrhea hospitalizations have been documented in many countries [1].

The World Health Organization (WHO) recommends the use of rotavirus vaccines in routine immunization programs worldwide [2]. However to date, the vaccines have not been introduced into the national immunization programs (NIP) of countries in Asia and only to a limited extent in Africa despite the high burden of severe rotavirus-associated diarrhea in these continents. In Indonesia, the rotavirus vaccine is recommended but is not publicly funded; therefore vaccine uptake depends largely upon whether healthcare providers (HCPs) recommend it to parents and its affordability.

To assist policymakers in deciding whether the vaccine should be introduced into a national program, it is important to get an understanding of the perspectives of consumers and providers’, as well as an understanding of their acceptance towards it use and any perceived issues. The importance of gathering this information is supported by the WHO guidelines for vaccine introduction which stresses that ‘the perceptions of the public and the medical community about the disease and the vaccine should be an important factor in determining its priority’ [3]. Given the ongoing call to include rotavirus into national vaccination programs, a program of research was instigated in Yogyakarta, Indonesia to examine the knowledge, perceptions, and attitudes of community members, HCPs and religious/community leaders toward rotavirus and the vaccine. This study reports on the findings from the HCP interviews.

## Methods

### Study design and sample

In-depth interviews were conducted between August and October 2013 targeting a range of HCPs in two districts of Yogyakarta Province. The cities of Yogyakarta and Sleman were chosen as they represented an urban and a rural area. Ethics approval was sought and received from the Human Research Ethics Committees at the Universitas Gadjah Mada (UGM) and from the University of New South Wales (UNSW), Sydney, Australia.

### Participants

Health providers were purposively selected to increase the richness of the information collected during the interviews: nurses, midwives, primary care providers and pediatricians. Participants were required to be aged 18 years and older and to be able to provide informed consent. Potential participants were recruited from two primary healthcare settings, from one private practice and from two hospitals (one academic teaching hospital and one private hospital). In addition, senior members of a number of professional health provider associations (including midvives and pediatric nursing) and health department members were also invited.

### Data collection

The authors, based on a review of the literature, developed an interview guide. Questions related to the following topics: (1) knowledge and attitudes towards diarrhea; (2) perceptions and experience with vaccines included/not included on the national immunization program (NIP); (3) knowledge of rotavirus diarrhea and vaccine; (4) acceptance of the rotavirus vaccine; (5) barriers impacting vaccination and (6) information needs and strategies for the provision of information. After ascertaining whether the participant had any baseline knowledge about rotavirus, a brief explanation regarding the epidemiology of rotavirus diarrhea was provided. In addition, a statement about the vaccine production process was also provided to participants.

Paraphrasing and additional questions were added to seek clarification during the interviews. Prompts were only given when the interviewer deemed that it is necessary to encourage the conversation back to topic or to address a certain issue. During the interviews, member checking was conducted to ensure rigor of research and that participants views were accurately reported [4]. This involved summarizing and feeding back participants’ views at the end of the interview to compare the investigator’s account with participants’ views. We continued with recruitment until we reached data saturation, where no new ideas or issues were raised in subsequent sessions [5]. Interviews were conducted by researchers from Universities Gadjah Mada in the local language.

### Analysis

All interviews were audio recorded, transcribed verbatim, translated into English and analyzed thematically. Transcripts for each taped interview were checked for internal consistency and corroborated with other interviews. The researchers used NVivo 10 for the analysis [6]. Following repeated and close reading of the individual interview transcripts, the researchers independently constructed a code list of major themes emerging from the data. These code lists were compared and crosschecked and a final list was compiled. An agreed thematic framework was then applied to another subsample of transcripts and further modified. Using this final framework, all of the 14 transcripts were analyzed and coded.

## Results

A total of 14 interviews were undertaken with three pediatricians, four primary care providers, four midwives, and three nurses. Participants worked in both public and private settings and had a range of experience in the provision of healthcare ranging from three to over twenty years. The interview results are presented thematically below.

### Not the number one problem

Participants acknowledge that diarrhea was an important health issue in Indonesia; however they did not rate it as the number one health problem. Instead they spoke of tuberculosis, respiratory illnesses, leptospirosis, and diabetes as being the high priority diseases. Episodes of diarrhea were associated with poor hygiene and sanitation, waste on the street and lack of clean drinking water. It was acknowledged that rotavirus affects people at every socio-economic level.

Some participants felt that with the correct treatment (i.e. rehydration, zinc), diarrhea was not considered dangerous. However, it was acknowledged that it could be life threatening if not managed promptly. Amongst the general public, the level of knowledge about dealing with diarrhea was assumed to be good, however it was suggested that parents don’t always have the financial capacity to take their children to a physicians and they may only see a midwives which could result in the child not receiving the appropriate level of treatment/care.

*“…Diarrhea can be serious if the treatment is incorrect but the main point is you have to do rehydration therapy. For me, any kind of diarrhea is not that dangerous as long as we can provide rehydration therapy” (Midwife)*

When asked about the strategies to prevent diarrhea, eating healthy food, breastfeeding, living well, and adhering to good hygiene practices were proposed. Very few spoke about vaccination as a preventative strategy. However, the general notion that prevention was better than treatment was voiced by some of the participants.

### Low levels of awareness and knowledge

Amongst the midwives and nurses interviewed there was very little knowledge about rotavirus and/or the vaccine. One of the nurses reported that she has not previously heard the word ‘rotavirus’ until described by the interviewer. In regards to the vaccine, other midwives and nurses reported their knowledge to be limited and all they knew was the vaccine was used to prevent diarrhea caused by rotavirus. All of the physicians reported that they knew about the pathogen and the vaccine. According to the HCPs interviewed, the community also has very little knowledge and/or awareness about the pathogen and the availability of the vaccine. It was suggested that further training be given to primary care providers and midwives in order to clinically identify and differentiate rotavirus from other causes of diarrhea and information on how to manage it accordingly.

### Mixed feelings towards the use of the vaccine

While most of the physicians believed that the vaccine was important and cost effective when compared to current treatments, some of the nurses/midwives felt that it would be better to use other approaches such as improved hygiene and access to clean water.

*“Actually diarrhea can be prevented, right, do not have to be given the vaccine. It is the factors of hygiene normally, so I think it is not very important as long as we are clean” (Nurse)*

The fact that the vaccine had not been included on the NIP led some people to believe that the Indonesian government also questioned the importance of vaccinating the community against rotavirus.

*“If the vaccine is not included in national programs yet, it must be not too important. Because the government should include the important things first into the program” (Midwife)*

Participants proposed that the vaccine could be recommend to ‘rich people’ so they can purchase it, while lower socioeconomic groups could be educated about cleanliness. Overall, it was suggested that members of the public would accept the vaccine as long as it was on the NIP, with the rational that members of the public are usually very welcoming of new vaccines on the NIP.

### The vaccine is too expensive

The current cost of the vaccine was considered to be the biggest barrier in promoting the use of the rotavirus vaccine in Indonesia. Midwives and physicians thought that it would be difficult to persuade people to vaccinate their children if the vaccine was not affordable. Vaccine cost would mostly impact those in the low socioeconomic levels as people in the middle and higher income groups would be able to afford the vaccine. One physician felt that the government hadn’t considered the vaccine affordability or a strategy for communicating the need for the vaccine to the general public.

*“Usually they will be happy when they know the purpose but then when they found out about the price, they will be sad. So usually they won’t get it.” (Midwife)*

*“They actually think about the cost more than anything here. If it’s for free, I’m sure they will get it but if they have to pay, they will rethink on it, even though they know it’s good for their children”. (Pediatrician)*

However, not all participants agreed that price of the vaccine was a big issue as reflected by the words of a pediatrician below:

*“Price in my opinion is relative. If we educate the parents well, then even if it’s expensive, it will be just perfectly fine with them. But if we wrongly educate them, even though it’s cheap, people will not buy it. One proof is with DPT vaccine, it was R450.000 but now it is being used daily here and there are demands and the demands come from middle class. So I think it’s not a problem, depends on how we educate. The community does care about their children, if that’s what they need then no matter what they will get it. (Pediatrician)*

They suggested that including the vaccine into NIP could solve this problem.

### Issues impacting on acceptance

A lack of awareness about the potential seriousness of diarrhea, about rotavirus and the availability of the vaccine was also postulated as a barrier. It was suggested that not only do members of the community lack knowledge about the pathogen and the vaccine, but also health professionals are unsure. Amongst the midwives who reported being reluctant to recommend the vaccine, a lack of knowledge was cited as the primary reason for not recommending it. This is reflected in the comments made by a midwife:

*“I have no courage to recommend it. If the parents asked further, I’m afraid I can’t answer because I don’t understand it yet. But I have recommended them for MMR and Hib vaccine.” (Midwife)*

The majority of the participants noted the issue of the vaccine not being ‘halal’ (An Arabic word meaning lawful or permitted). Many accepted the need for the vaccine but highlighted that the vaccine would not be accepted as it is ‘haram’ (The opposite of halal is haram, which means unlawful or prohibited). Participants noted that the vaccine must have the official label from the Majelis Ulama Indonesia (MUI).

*“Principally, I agree with the immunization, but the problem is that during this time, the vaccine is contaminated with pig enzyme”. (Pediatrician)*

It was also suggested that members of the community downplay their religious concerns about the vaccine in order to avoid arguments with their physicians, instead focusing on other vaccine concerns.

*“When the problem is about religion, they often hide it, but they tend to talk about the side effect as they have read from the Internet. They don’t want to debate about belief to the physicians” (Pediatrician)*

### Provider barriers to vaccination

From a provider perspective, participants suggested that health professionals are too busy to promote the vaccine and that during the consultations only has the time to focus on discussing the NIP vaccines with parents. It was also postulated that access to the vaccine in remote areas is a problem affecting the use of the vaccine.

“*We have too many patients to be immunized in a single day (50–60 patients), so there is no time”. (Midwife)*

*“service is not available everywhere. So if their house are far away then they have to make effort just to go to PHC is difficult decision for the parents to vaccinate their children.” (Midwife)*

Lastly, having sufficient quantities of vaccine (preferably locally manufactured) was also highlighted as being important.

*“The number one obstacle is production, where we are not able to produce it yet. So like in the case of polio, we did not have any until half a year and that’s a huge obstacle in immunization. So if we do wish to put it in the government’s program, make sure the production is good, better if we can make it on our own”. (Pediatrician)*

### Important to work with local leaders and to use mass media

The need to work with local public health officers, religious leaders and local government leaders to promote the need for the vaccine was highlighted in most of the interviews. One pediatrician suggested that it would be a good idea to take the religious leaders through the vaccine manufacturing process, so that they can issue a certificate which states that the vaccine is ‘halal’ in order to make it acceptable to patients. One participant even went as far as to suggest that the Sultan (King and Governor of Yogyakarta) should be involved.

*“The involvement of the mayor, regent, headman* etc. *is extremely important. I wish the Sultan would just say that all the citizens are required to be vaccinated with the rotavirus vaccine. I’m sure no one will refuse, especially if someone attends him from the MUI who explains about the halalness of this vaccine and also someone from the drug company making this vaccine and also a physician who explains about the danger from rotavirus infection. I’m sure no one within the community will dare to refuse, but no such thing ever happens” (Pediatrician)*

*“The capacity to explain and the tupoksi (responsibility) are with the health service office, puskesmas, then the cadres or public figures. Those cross-sectorial people should be involved here. So, we cannot work alone; it is difficult” (Physicians)*

Advertising on local television and in local newspapers was suggested as the most effective approaches for reaching people in remote areas. In addition, it was proposed that seminars and counselling at mothers meetings be conducted to educate people about the vaccine. Given that not all members of the community are literate, it is important that local health cadres also verbally promote the messages about the disease and the vaccine.

## Discussion

From the interviews we identified that diarrhea is not always rated as an important problem in Indonesia, but participants do acknowledge that it can be serious if not properly treated. While there was some level of knowledge about rotavirus, very few participants knew that a vaccine was available. Lastly, there were some who believed that vaccination against rotavirus was unnecessary given the fact that there are alternative approaches to preventing diarrhea.

In 2007, Simpson et al. undertook interviews with public health providers about in rotavirus in five low- and middle-income countries including Indonesia [7]. In corroboration with our study, they also reported that in all five settings, knowledge of rotavirus was extremely low and their participants also ranked the disease as not being a high priority. In Indonesia, the rotavirus vaccine is currently only available in pediatricians’ private practices. It is therefore not unexpected that the midwives participating in our study had very little knowledge and awareness about the vaccine given that they all worked in public primary health centers. Amongst the midwives who reported being reluctant to recommend this vaccine they cited lack of knowledge as the main reason.

Mixed levels of support for the rotavirus vaccine have been reported in high-resource settings [8, 9]. In low/middle income settings, very few studies have been undertaken to examine the attitudes of HCPs towards the use of the rotavirus vaccine. One survey of Indian pediatricians identified that over 70 % are using the rotavirus vaccine selectively, while only 9.7 % administer it routinely and 16 % do not administer it at all. Interestingly, only 62.1 % of their participants believed that rotavirus disease would be severe in an infant and only 24.3 % felt it would be severe in a child aged 1–5 years. Lastly, only 45 % reported that they believed that the rotavirus vaccine was efficacious [10]. This is an important finding given the fact that the attitudes of health professionals are major factor associated with vaccine uptake [11]. In order to promote the use of the vaccine, communication messages need to be clearly defined and directed. Information must be provided to all types of HCPs including midwives, nurses and physicians about the burden of disease, the epidemiology of rotavirus in Indonesia, the cost of disease vs. the cost of vaccination, safety and effectiveness associated with the vaccine and the rationale for vaccination. It is important that the education messages break down the misconceptions around diarrhea and rotavirus especially. For example, we found that participants believed that improved hygiene and cleanliness would prevent cases of rotavirus diarrhea. However, we know rates of rotavirus diarrhea are universally similar (pre-vaccine) indicating that hygiene practices do not reduce transmission of rotavirus.

While the attitudes of providers and parents will impact on the use of the vaccine, one of the biggest barriers is ultimately the cost of the vaccine. In Indonesia the price of the vaccine is between US$17–$23 per dose on the private market. While this is not the most expensive vaccine available on the private market (Pentavalent DTPa-HBV-Hib and pneumococcal vaccines cost between US$30–$58 per dose), it is unlikely that some community members will opt to purchase this vaccine. Participants proposed that the vaccine needs to be added to the NIP and then both community members and HCPs would accept it. The most effective way to ensure maximum uptake of vaccination is to provide reimbursement for the vaccine within a NIP [12].

A 2013 Indonesian economic evaluation indicated that the rotavirus vaccination program would be cost-neutral at a price of US$2.39–3.01 (health-care perspective) to US$3.45–4.13 (societal perspective). If the rotavirus vaccine could be administered for US$2.70 or less per course it would be potentially cost saving from the Indonesian health-care perspective and at US$3.79 or less per course as cost saving from a societal perspective. Even at a price of US$ 14 per course, rotavirus immunization would be highly cost-effective given Indonesia’s GDP [13].

Indonesia has the largest Muslim population of any country—212 million of its 241 million people (88 percent) [14]. Some members of Islamic faith may object to receiving the rotavirus vaccine, arguing that it is “haram” (not permitted) as porcine products are used in the manufacturing process as highlighted by our participants. However, scholars of the Islamic Organization for Medical Sciences in conjunction with the WHO have determined that the transformation of pork products into gelatin sufficiently alters them, thus making it permissible for observant Muslims to receive vaccines containing gelatin [15]. In Indonesia, the primary Muslim groups including members from Majelis Ulama Indonesia (Indonesia’s top Muslim clerical body), Muhammadiyah and Nahdlatul Ulama (Independent Islamic organizations), attended a workshop in 2011 and agreed that the rotavirus vaccine should be considered as halal. Despite this, the vaccines currently available in Indonesia are yet to receive the official symbol on the label. Participants reported that lengthy explanations about the production of the vaccine are unnecessary as parents are happy enough to just be shown the symbol on the vial. While there is uncertainty whether this will happen, HCPs should be reminded that the rotavirus vaccine is routinely used in many other majority-Muslim countries including Malaysia, Bangladesh, Saudi Arabia and Egypt [16]. In addition, the rotavirus vaccines available in Indonesia, the monovalent human rotavirus vaccine (RotarixTM, GlaxoSmithKline), received halal certification in June 2014 from the European Union Halal Food Council of Europe (HFCE), while the bovine backbone vaccine (RotateqTM, Merck &Co) received halal certification in in 2013 by the Islamic Food and Nutrition Council of America (IFANCA).

The use of in-depth interviews to elicit a greater depth in the information is a key strength of our work. However, we acknowledge that there are limitations associated with our work. Firstly, interviews were only undertaken with a small select group of HCPs from two selected geographic locations, so the possibility of other important themes emerging cannot be ruled out. The application of our findings to a broader population may be limited due to the unique characteristics of our study sample. This may have also been heightened by the fact that some of the participants were unaware of the vaccine prior to their involvement in the study. Large quantitative surveys should be undertaken with a representative sample of HCPs to verify the findings from our study. The use of in-depth interviews are an important way of identifying important issues around the use of the rotavirus vaccine and our study has shed further light on the barriers to rotavirus vaccination.

## Conclusion

The current cost of the rotavirus vaccines in the private market and the low priority given to this vaccine are the biggest obstacles impacting on the adoption of this vaccine in Indonesia. Disease burden is universally considered to be an important driver of decisions to adopt new vaccines. HCPs need to be reminded of the burden of disease associated with rotavirus, the costs coupled with treating cases as compared to the costs associated with preventing cases may also assist with improving acceptance towards the vaccine. In addition, providers and consumers need to be reminded that there are no remnants of porcine products remaining in the final vaccine. Promotion campaigns need to target the range of HCPs involved with providing care to pregnant women and young children.
